# The Clinical Significance of CD163+ Tumor-Associated Macrophages (TAMs) in Laryngeal Squamous Cell Carcinoma

**DOI:** 10.7759/cureus.36339

**Published:** 2023-03-18

**Authors:** Abderrahman Ouban, Emadeddin Raddaoui, Mohamad Bakir

**Affiliations:** 1 Department of Pathology, College of Medicine, Alfaisal University, Riyadh, SAU; 2 College of Medicine, Alfaisal University, Riyadh, SAU

**Keywords:** laryngeal pathology, laryngeal tumors, tissue microarrays, laryngeal squamous cell carcinomas, tumor associated macrophages (tams), cd163

## Abstract

Background and objective

The tumor’s microenvironment is currently considered an important indicator of the tumor’s prognosis, treatment failure, and recurrence. CD163+ tumor-associated macrophages (TAMs) are a marker of poor prognosis in many types of human cancers. In the present study, the expression of CD163+ TAMs was analyzed in laryngeal squamous cell carcinomas (LSCCs) using immunohistochemistry, and this expression was correlated with the clinical and pathological characteristics of LSCC patients.

Materials and methods

One commercial human larynx microarray with 80 cases of LSCCs, was used for this study. For comparison with normal laryngeal mucosa, a second microarray carrying normal tissues from all human anatomical sites, including normal laryngeal tissues, was used. Immunohistochemical staining was performed, and the primary antibody was a mouse monoclonal against human CD136. The absence of the primary antibody was used as a negative control. The percentage of positive cells was categorized into five scores: 0 (0%); 1, (1%-10%); 2, (11%-50%); 3, (51%-80%); and 4, (>80%). A case was scored as positive for CD163 with a score >= 1. The χ2 test was used to assess the CD163 expression in LSCC cases (N=80). A statistically significant difference was defined as P 0.05.

Results

The human larynx microarray containing 80 cases of LSCCs was used for this study. The age of the cancer patients in this array was in the range of 39 to 72, with a median of 53. LSCC grades were distributed as follows: 25 patients were designated as grade I, 43 were designated as grade II, and 6 were designated as grade III. Two tumors' (2/80) cores were missing from the microarray. Six tumors on the microarray did not have a grade designation reported by the manufacturer of the array. The expression of CD163 in normal, benign, unmatched laryngeal tissue was absent. In cancer cases, on the other hand, a significant number of LSCCs had TAMs that were positive for CD163 (87% positive tumors, with an IHC score ranging from 1 to 4, χ2=30.634; p<0.001). The rest of the LSCC cases (10 in total) had negative CD163 expression (score of 0).

Conclusion

A significant majority of LSCCs were found to have CD163+ TAMs expression using tissue microarrays (TMAs). This expression is positively correlated with the tumor’s grade, clinical manifestation, and TNM staging. Morphologic evidence shows that the majority of LSCCs express the highest range of immunohistochemistry (IHC) scores for CD163 protein in the membranes and cytoplasm of their TAMs. This study provides evidence of the clinical significance of CD163+TAMs in LSCCs and proposes further studies to pinpoint the exact role of these cells in LSCC patients.

## Introduction

Squamous cell carcinoma of the larynx is the second most common cancer in the head and neck region, making up 30% of tumors in that site [1]. Novel treatment modalities such as chemotherapy, radiation, and surgical procedures have not improved the survival rates of patients with high-stage laryngeal cancer in the past 30 years [1]. With a five-year survival rate of less than 30% [2], patients with advanced-stage laryngeal cancer still face a 40%-50% likelihood of tumor recurrence or metastasis [2]. There is a great need for new biomarkers of prognosis and management for this subset of head and neck cancer. For example, a recent report pointed out leptin as a valuable biomarker of laryngeal cancer recurrence following treatment [3]. More recent reports implicate Filamin-A (FLNA) expression in cell migration, progression, and prognosis of laryngeal squamous cell carcinoma (LSCC) [4-6]. In the recent literature, evidence is mounting regarding the important role of the tumor’s microenvironment in its progression and prognosis [7,8]. The tumor’s microenvironment is made up of extracellular matrix and immune cells, prominently among which are tumor-associated macrophages (TAMs), also known as M2 cells [7-10].

Not only do TAMs have poor antigen-presenting capabilities, but they also produce factors that suppress the normal immune regulatory functions of T-cells [7]. In contrast to their normal counterparts, M2 macrophages from experimental or human tumors are incapable of lysing tumor cells or supporting the anti-tumor functions of T cells and natural killer (NK) cells in vitro [8]. These cells have lost their normal functions after being recruited and co-opted by the tumor through exposure to cytokines produced by the tumor cells, such as IL-4, IL-10, transforming-growth factor-β1 (TGF-β1), and prostaglandin E2 (PGE2) [9]. The newly altered functions of M2 cells include modulating the inflammatory response, scavenging cellular debris, promoting angiogenesis, and tissue remodeling, the latter two functions being essential for creating the microenvironment of the tumor [7-10]. Their functions also include promoting tumor cell invasion and metastasis to local and distant sites [7-11]. As evidenced by the role played by M2 cells in cancer, knocking down the CSF-1 gene to deplete macrophages in a mouse model of breast cancer resulted in a slower progression from pre-invasive lesions to full-blown invasive cancers, a steep reduction in tumor growth, and an overall reduction in metastases [12,13]. TAMs' role in cancer development and progression is further supported by evidence linking these cells to angiogenesis, which is seen in many cancers, including breast cancer, cervical cancer, follicular lymphoma, prostate carcinoma, renal carcinoma, and esophageal carcinoma, inferring a poor prognosis for those tumors [14-22]. While the expression and role of TAMs have been studied in different types of human cancers, there is a deficiency in studies analyzing this expression and its correlation with laryngeal cancer. Furthermore, almost all of the above-mentioned studies were performed utilizing classic, full histopathological sections. Selection bias may be introduced when utilizing such tissue samples, as it involves the selection of areas within slides to average out the intensity of the TAMs signal. By using tissue microarrays (TMAs) in this study, no selection bias will be introduced, as the signal of TAMs seen will be reported in total.

## Materials and methods

Tissues

One commercial human larynx microarray (catalog no. LP803; Biomax US, Rockville, MD), with 80 cases of LSCCs, was used for this study. All the cases were confirmed as LSCCs. Major parameters of these tumors include age, sex, anatomical site, pathological diagnosis, grade, clinical, and TNM staging. For comparison with normal laryngeal mucosa, a second microarray (catalog no. FDA 992, Biomax US, Rockville, MD) carrying normal tissues from all human anatomical sites, including normal laryngeal tissues, was used. A normal benign tonsillar tissue obtained from an unrelated patient was used as a positive control for FLNA expression.

Immunohistochemical staining

Immunohistochemical staining was performed following deparaffinization and antigen retrieval with the UltraView Universal DAB Detection Kit (v1.02.0018, Venata Medical Systems, Basal, Switzerland) using the BenchMark Ultra immunohistochemistry/in situ hybridization staining module (Venata Medical Systems). The TMAs were incubated with the primary antibody for 32 minutes at 37 °C. The primary antibody, which was diluted as per specific suppliers’ instructions, was a mouse monoclonal against human CD163 (MRQ-26, 760-4437-Roche Diagnostics, Indianapolis, IN). The absence of the primary antibody was used as a negative control. Positive control of normal tonsillar tissue was used for test optimization and to run validation.

CD163 IHC Evaluation

Immunohistochemical staining of CD163 for all tumors was evaluated by two pathologists (AO) and (ER), independently, and a consensus was reached for discordant cases. Positive cells for CD163 expressing a brown or black stain in their membrane or cytoplasm were identified as TAMs (M2) [23, 24]. Because the study is analyzing TAMs in core representative sections of a TMA, the CD163+ TAMs infiltrating the epithelium and stroma were estimated in total. While other studies performed on classic, full histopathology slides resorted to identifying three or more representative sections where TAMs counts were done, then averaging them out and using that average for statistical analysis [22,25], the TMA's precise, representative sections allow for a more precise, less subjective estimate of the total number of TAMs in that section. Consequently, a modified version of Baccelli et al.'s protocol is used to count TAMs in TMA core sections [26]. The percentage of positive cells was categorized into five scores: 0 (0%); 1 (1%-10%); 2 (11%-50%); 3 (51%-80%); and 4 (>80%). A case was scored as positive for CD163 with a score >= 1.

Statistical analysis

The χ2 test was used to assess the CD163 expression in LSCC cases (N=80). The χ2 test was also used to examine the relationship between CD163-positive cases (N = 68) and various clinical, demographical, and pathological criteria (age, gender, tumor grade, clinical and TNM staging). All statistical analyses were performed with the IBM SPSS Statistics software package, version 25.0. P<0.05 was considered to indicate a statistically significant difference.

## Results

The human larynx microarray (catalog no. LP 803, Biomax, US, Rockville, MD) containing 80 cases of LSCCs was used for this study. The age of the cancer patients in this array was in the range of 39 to 72, with a median of 53. LSCC grades were distributed as follows: 25 patients were designated as grade I, 43 were designated as grade II, and 6 were designated as grade III. Two tumors' (2/80) cores were missing from the microarray. Six (6) tumors on the microarray did not have a grade designation reported by the manufacturer of the array. The TNM and clinical stage were assigned according to the American Joint Committee on Cancer; Cancer Staging Manual, 8th edition [27]. All patients’ tumors in the microarray have clinical stages and TNM designations, as reported by the manufacturer of the array.

**Figure 1:**
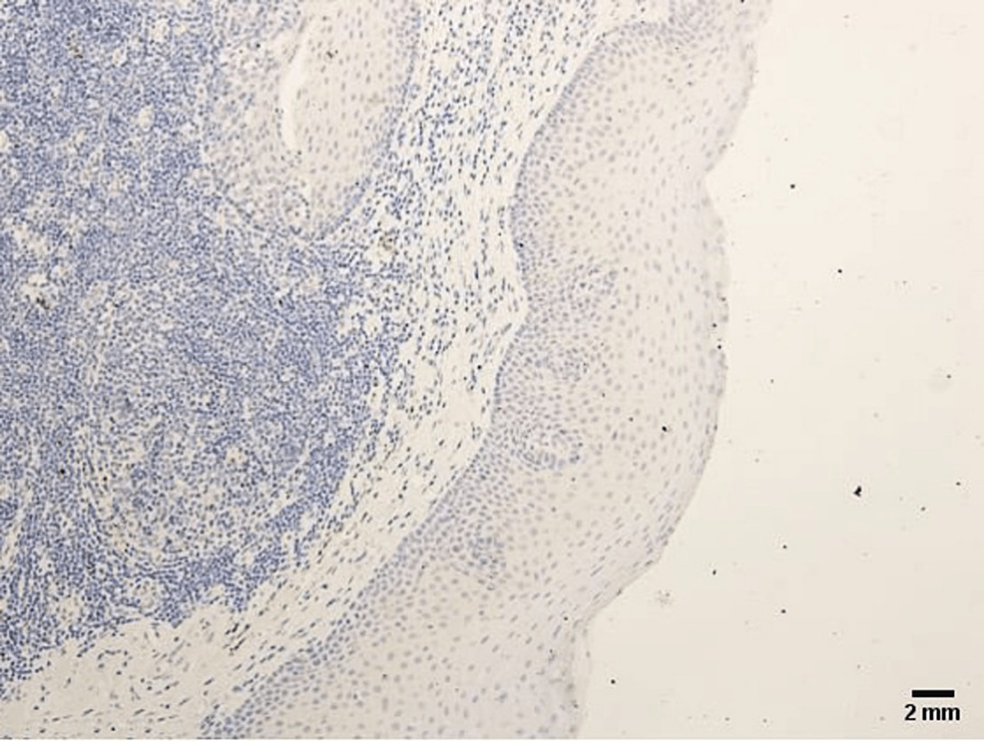
CD163+ TAMs expression in normal, benign, unmatched laryngeal tissue is absent. 2 mm scale bar.

The clinical stages were distributed as follows: eight patients were designated as stage I, 29 patients were designated as stage II, 32 patients as stage III, and 11 patients as stage IV. The Ts (in reference to primary tumors in TNM) were distributed as follows: eight patients were designated as T1, 40 patients were designated as T2, 21 were designated as T3, and 11 were designated as T4. The Ns (in reference to lymph node status in TNM) were designated as follows: 60 patients were designated as N0, 19 as N1, and 1 as N2. The Ms (in reference to distant metastases in TNM) were distributed as follows: 80 patients were designated as M0. The designation of TNM is as follows [27]: T represents the primary tumor, with subsets T1 (tumor invades submucosa), T2 (tumor invades muscularis propria), T3 (tumor spreads into the subserosa via the muscularis propria), T4 (tumor directly invades other organs or structures and/or perforates visceral peritoneum); N represents the regional lymph nodes, with subsets N0 (no regional lymph node metastasis), N1 (metastasis in 1 to 3 regional lymph nodes), N2 (metastasis in 4 or more regional lymph nodes), N3 (metastasis in 10 or more regional lymph nodes, nodes under the clavicle or collarbone, or internal mammary nodes); M represents distant metastasis, with subsets M0 (no distant metastasis), M1 (distant metastasis). The expression of CD163 in normal, benign, unmatched laryngeal tissue (TMA with Catalogue No. FDA 992, Biomax US, Rockville, MD) was absent (Figure 1).

**Figure 2:**
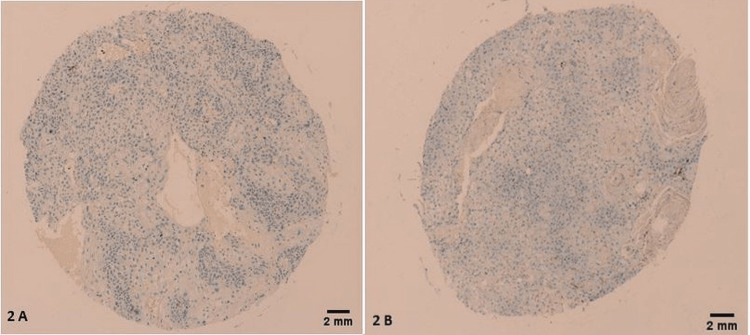
Negative CD163+ TAMs expression was found in a few tumors in the study sample. (A, B) Tumors are both grade 2, stage II, T2N0M0. The scale bar is 2 mm.

In cancer cases, on the other hand, a significant number of LSCCs had TAMs that were positive for CD163 (87% positive tumors, with an IHC score ranging from 1 to 4, χ2=30.634; p<0.001, Table 1). The rest of the LSCC cases (10 in total) had negative CD163 expression (score of 0; Figures 2A, 2B).

**Figure 3:**
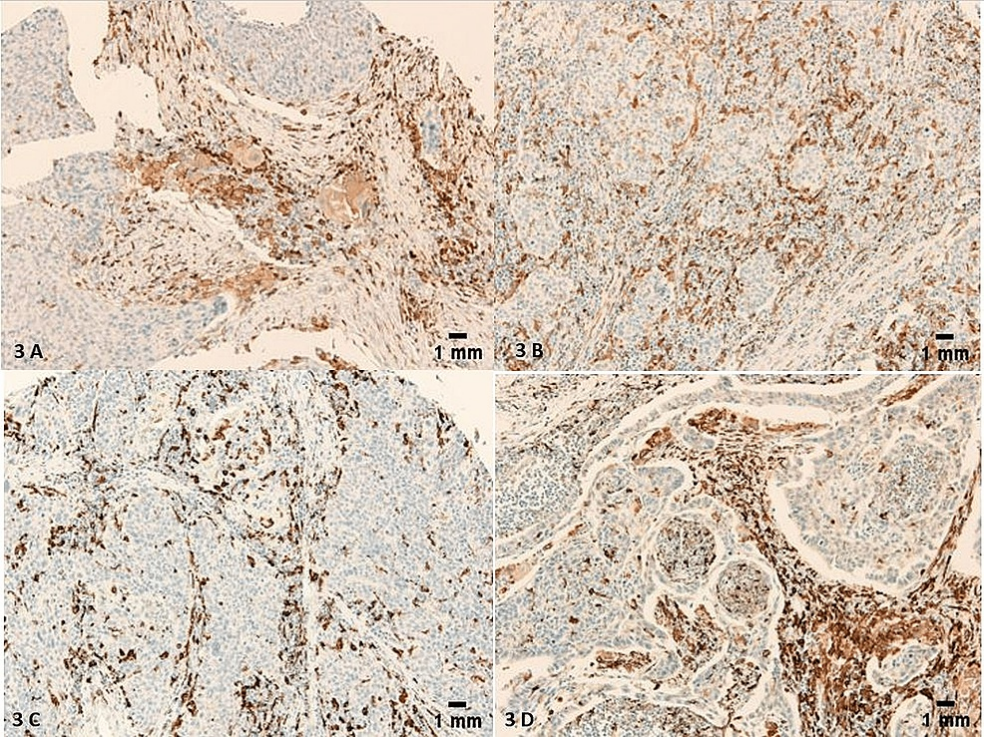
Membranous and cytoplasmic staining of TAMs with CD163. No nuclear staining was identified in TAMs. (A-D) Scale bar = 1 mm.

All positive LSCC cases (Table 1) expressed the marker in the membrane and the cytoplasm of the TAMs (Figures 3A-3D).

**Table 1:** CD163 expression in TAMs present in laryngeal squamous cell cancers and its subcellular location. Chi-square analysis was used to assess the CD163 expression in TAMs and to examine its expression location within those cells (cytoplasmic/membranous vs. nuclear). NEG, negative; POS, positive. *: indicates a statistically significant result.

CD163 expression and position in TAMs	All cases, n	%	FLNA protein expression		
						-	-	-	x^2	df	P-value
CD163 Expression			30.634	1	<0.001*
NEG	10	13	-	-	-
POS	68	87	-	-	-
Subcellular localization of CD163			-	-	-
Nuclear	0	-	-	-	-
Cytoplasmic/Membranous	68	100	-	-	-

Association between CD163 expression and clinical-pathological parameters in LSCC cases

The association between CD163+ TAMs expression and clinical-pathological parameters was further analyzed in the LSCC cases (Table 2). Positive staining for CD163+ TAMs was significantly correlated with LSCC grade (χ2=24.92; p<0.001); clinical stage (χ2=12.949; p=0.0047), primary tumor status T (χ2=16.70; p=0.0008); lymph node status N (χ2=44.05; p<0.001) and metastatic status M (100% of M0). There was no significant association between CD163+ TAMs expression and age of the patients (p=0.928) or sex of the patients (p=0.809).

**Table 2:** Clinical, pathological, and demographic characteristics of FLNA-positive cases. Chi square analysis was used to examine the relationship between FLNA-positive cases and gender, age, differentiation grade, clinical, and TNM staging. G1, grade 1; G2, grade 2; and G3, grade 3. *: indicates a statistically significant result.

Clinical/Path	All cases, n	%	Significance		
						-	-	-	x^2	df	P-value
Gender			-	-	-
Male	61/69	88.40	0.058	1	0.809
Female	7/9	77.70	0.058	1	0.809
Age			-	-	-
>53	46/54	85.1	0.008	1	0.928
<53	22/25	88	0.008	1	0.928
Stage			-	-	-
I	7	8.75	12.949	3	0.0047*
III	23	28.75	12.949	3	0.0047*
III	27	33.75	12.949	3	0.0047*
IV	11	100	12.949	3	0.0047*
T			-	-	-
T1	6	8.82	16.7	3	0.0008*
T2	31	45.59	16.7	3	0.0008*
T3	20	29.40	16.7	3	0.0008*
T4	11	16.18	16.7	3	0.0008*
N			-	-	-
N0	52	76.50	44.05	2	<0.001*
N1	15	22.05	44.05	2	<0.001*
N2	1	1.53	44.05	2	<0.001*
M			-	-	-
M0	68	100	-	-	-
M1	0	-	-	-	-
Differentiation Grade#			-	-	-
W. diff. (G1)	18	26.47%	24.92	2	<0.001*
M. diff. (G2)	43	63.23%	24.92	2	<0.001*
P. diff. (G3)	5	7.35%	24.92	2	<0.001*

Table 3 provides a summary of the range of IHC scores in CD163 positive LSCCs.

**Table 3:** Range of IHC Scores in CD163 positive LSCCs

CD163 Positive LSCCs (N=68)	IHC Score of CD163	Significance		
-	-	x^2	df	P-value
21 LSCCs	4	26.488	3	p<0.001
33 LSCCs	3			
13 LSCCs	2			
1 LSCCs	1			

**Figure 4:**
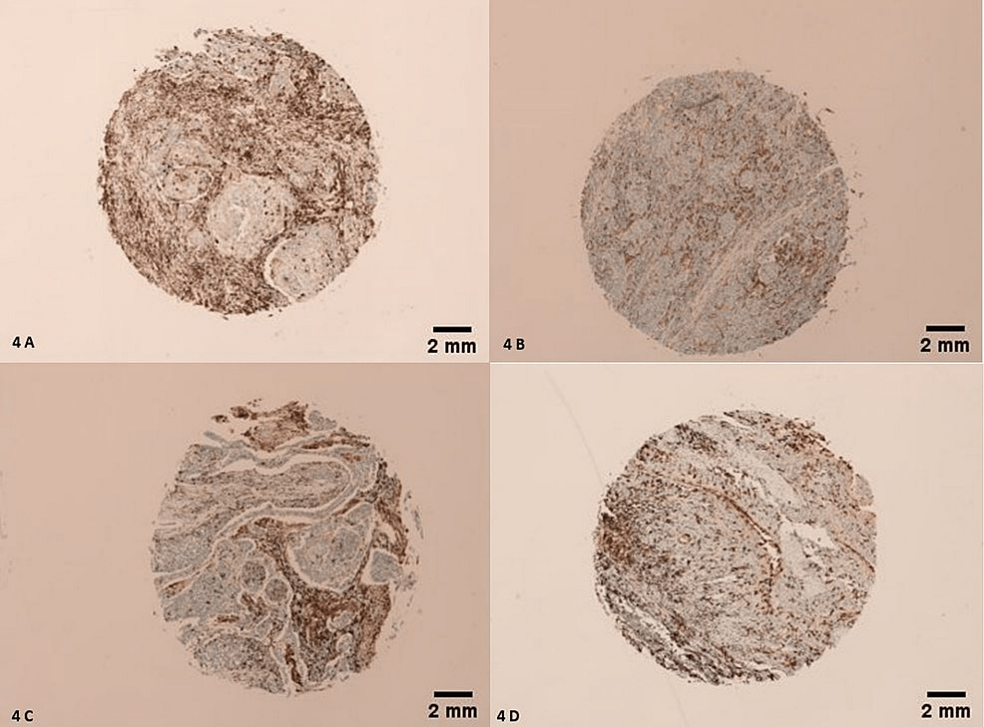
Heavy membranous and cytoplasmic staining of TAMs by CD163. IHC score of 4/4. (A, B) Tumors are grade 2, stage III, T3N0M0. (C) Tumor is grade 2, stage IVA, T4aN0M0. (D) Tumor is grade 2, stage III, T3N0M0. Scale bar = 2 mm.

**Figure 5:**
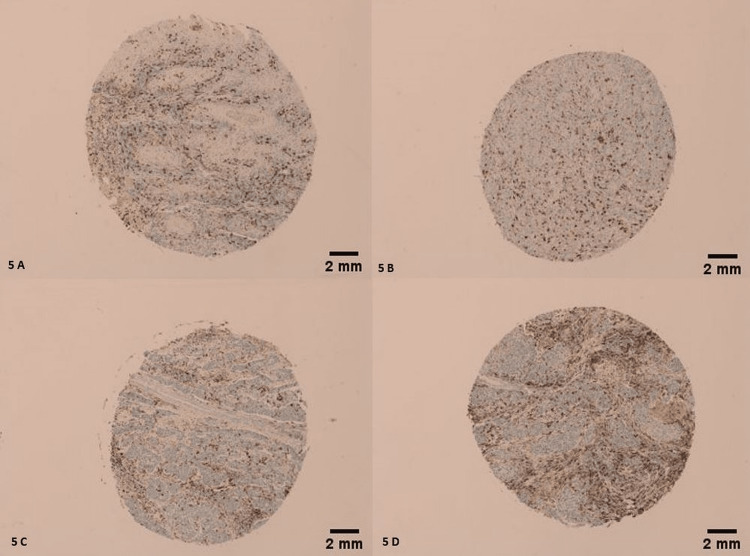
Moderate membranous and cytoplasmic staining of TAMs by CD163. IHC score of 3/4. (A) Tumor is grade 2, stage II, T2N0M0. (B) Tumor is grade 3, stage IVA, T4aN0M0. (C, D) Both tumors are grade 2, stage II, T2N0M0. Scale bar = 2 mm.

**Figure 6:**
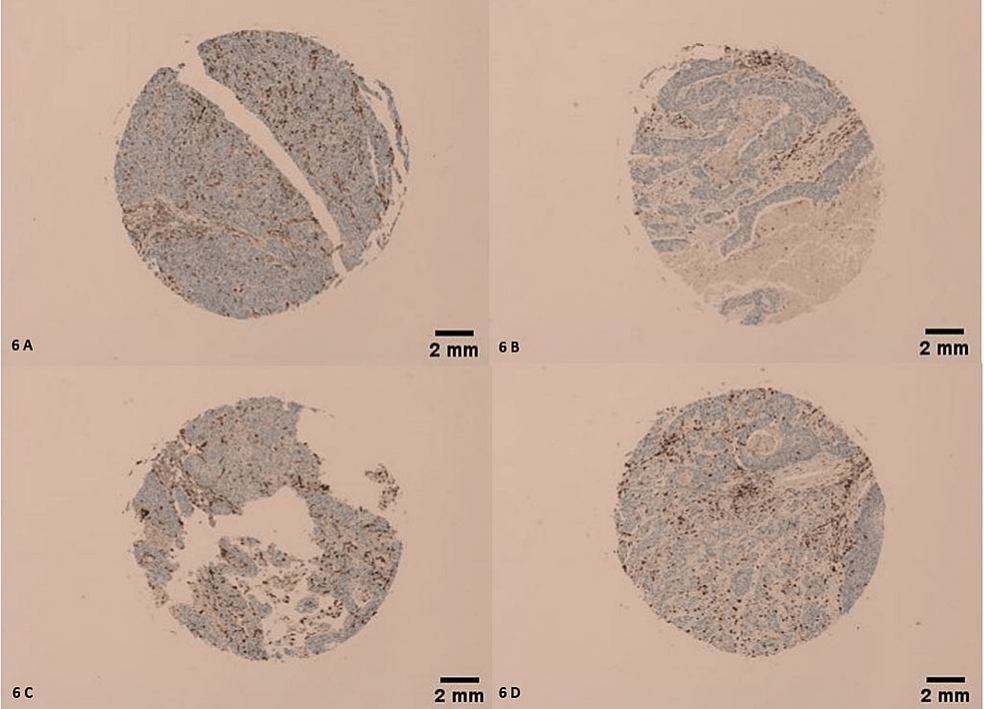
Mild membranous and cytoplasmic staining of TAMs by CD163. IHC score of 2/4. (A) Tumor is grade 2, stage II, T2N0M0. (B) Tumor is grade 1, stage II, T2N0M0. (C) Tumor is grade 1, stage III, T2N1M0. (D) Tumor is grade 2, stage II, T2N0M0. Scale bar = 2 mm.

**Figure 7:**
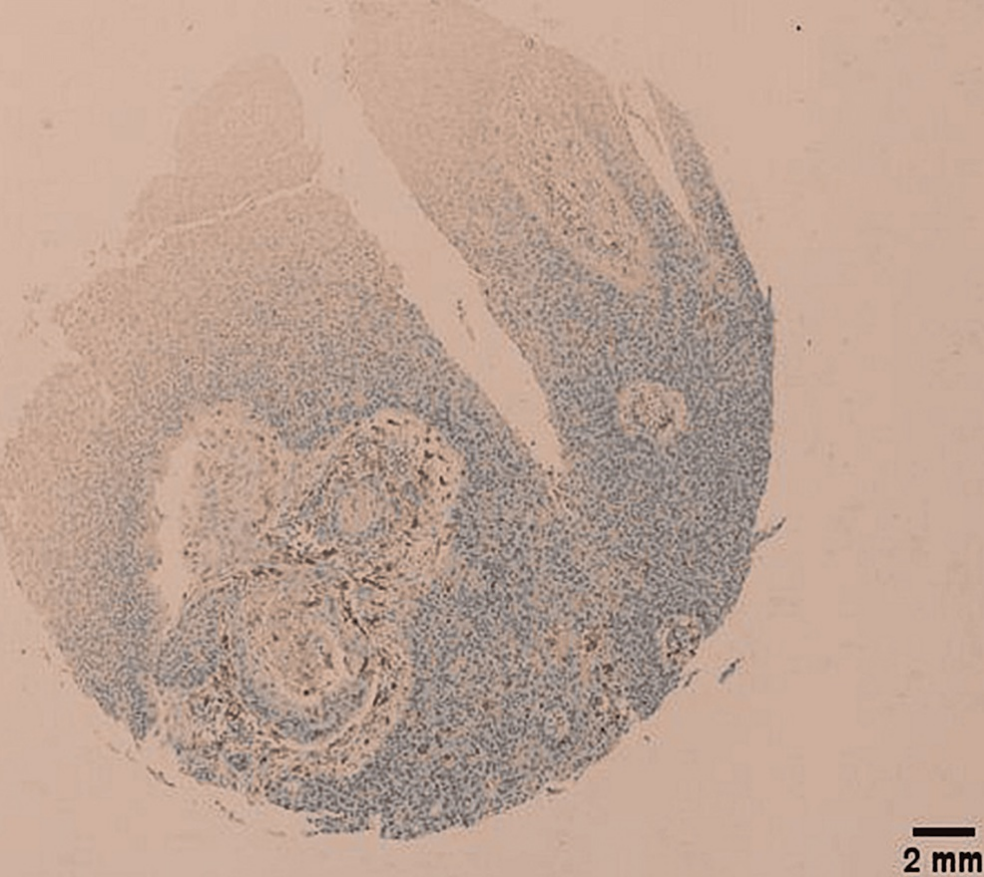
Mild membranous and cytoplasmic staining of TAMs by CD163. IHC score of 1/4. Tumor is grade 2, stage III, T3N1M0. Scale bar = 2 mm.

Morphologically, 21 CD163 positive tumors (21/68) had an IHC score of 4/4 (Figures 4A-4D), 33 CD163 positive tumors (32/68) had an IHC score of 3/4 (Figures 5A-5D), 13 of them (13/68) had an IHC score of 2/4 (Figures 6A-6D), and only 1 had an IHC score of 1/4 (Figure 7) (χ2=26.488; p<0.001). Only 10 LSCCs were negative (Figures 2A, 2B). All of the LSCCs’ TAMs expressed CD163 in the membranes and cytoplasm of its cells only, and no nuclear staining could be identified (Figures 3A-3D).

## Discussion

This study is the first to analyze the expression and distribution of CD163+ TAMs in LSCCs and to correlate this expression with the clinical-pathological attributes of these tumors. CD163+ TAMs were present in a significant number of tumors (p<0.001). TAMs express CD163 exclusively on their membranes and cytoplasm. CD163+ TAMs correlated with clinical stage, TNM staging, and the grade of the tumors (Table 2). Given the extensive reports linking TAMs with cancer in general [7-25], this study, which is the first immunohistochemical study analyzing CD163 expression by TAMs in these tumors using TMAs, confirms the above results.

The importance of this study’s findings emanates from the multiple roles the M2 (TAMs) cells play in carcinogenesis. Its versatile role encompasses tumor growth [28], neo-angiogenesis [22], blunted immune response [9,29], invasions [30,31], and establishing metastases [32,33].

The extensive involvement of these cells in carcinogenesis, as is evident from the above, may allow for a greater response when they are targeted, as they may exert their role at multiple points along the oncogenesis highway in the same tumor. At the same time, it may provide a therapeutic intervention that could be utilized in different types of malignant tumors where CD163+ TAMs are present. With the recurrence rate of LSCCs standing at 50% for advanced disease, i.e., T3 and T4 stages [2,34], it is imperative to investigate this expression as a possible tool in the management of LSCCs. Few studies have addressed the microenvironment of LSCCs. A recent study investigated the expression of CD163+ TAMs in tandem with KI67 and found that they could be used as diagnostic biomarkers to differentiate low-grade from high-grade dysplasia of the larynx [23]. The authors found that the number of CD163+ TAMs significantly increased when comparing high-grade vs. low-grade laryngeal dysplasia and carcinoma-in-situ vs. dysplasia. The study was performed on routine, full histological sections. No correlation analysis between CD163+ TAMs and clinical pathological attributes was performed [23]. A second study of supraglottic LSCCs used CD68 as a marker of TAMs. The authors found that supraglottic laryngeal carcinoma patients with high TAMs-infiltration were prone to metastases and had poor prognoses and low survival rates [25]. The results of this study are in line with the above, showing that a significant expression of CD163+ by TAMs is associated with a higher grade, clinical stage, and TNM stage. This is the first such study to be performed on TMAs. TMAs, by their design, allow for the evaluation of all the cells present rather than representative regions within the full histopathology slide, and that may introduce selection bias. This is also the first laryngeal study that uses CD163 to pinpoint TAMs in laryngeal cancer.

Regarding the exact role of these cells in LSCC, it is not known yet, however, a review of the role of CD163+ TAMs in other tumors may shed some light on its possible mechanism in LSCC. Early on in oncogenesis, it seems that tumor cells may invite macrophages, where they co-opt these cells and “re-educate” them to aid in the transformation process [7,22]. In return, these TAMs will promote cancer cell growth and expansion by producing several factors that are known to expand the malignant clone, such as the epidermal growth factor (EGF) [28,35], platelet-derived growth factor (PDGF) [36], transforming growth factor B1 (TGF-B1), hepatocyte growth factor, and basic fibroblast growth factor (b-FGF) [36]. On the other hand, there is considerable evidence indicating the importance of the role TAMs play in neo-angiogenesis. TAMs produce a number of essential proangiogenic factors, cytokines, and molecules, including bFGF, IL-8, TNF-alpha, and VEGF, which assist the malignant clone by providing it with its vascular network [22]. Besides the above, TAMs also express a broad array of angiogenesis-modulating enzymes, including matrix metalloproteinases (MMPs)-2, -7, -9, and -12 and cyclooxygenase-2 (COX-2) [36-38]. This large number of pro-angiogenic factors and enzymes produced by TAMs may explain the association seen between a high number of TAMs and high vascularity seen in many cancer subtypes, including esophageal, breast, urothelial, buccal, melanoma, glioma, and prostate carcinomas [18,20,32,33,39-41]. TAMs are also strongly related to tumor invasion, where there is a preponderance of evidence indicating that TAMs facilitate tumor invasion through the basement membrane and deep into soft tissue by being involved in the production or activation of several proteolytic enzymes and genes involved in this process, such as cathepsin B in macrophages [22], and MIF and extracellular MMP inducer (EMMPRIN) in tumor cells [30,31]. Finally, there is also solid evidence linking TAMs with the establishment of metastases, starting with the separation of individual cells from the original tumor mass all the way to the establishment of secondary tumors at distant sites [32,33,42]. This was evident when Gorelick and co-workers presented evidence that the movement of tumor cells away from the main primary tumor always happens with TAMs in the vicinity, and that reaching the blood by extravasation often occurs at the site where groups of TAMs are in position (attached) to the surface of the blood vessels [42]. It is of note that all the above is occurring while the immune system is suppressed, where TAMs are ensuring a blunted immune response against cancer cells. When the M2 macrophages are exposed to a variety of factors, cytokines and peptides produced by cancer cells, such as IL-4, IL-6, IL-10, TGF-B1, and PGE2, they tend to lose the ability for normal maturation [43], and the ability to produce free oxygen radicals [9,29], thus losing their cytotoxic activity [9,44] on one hand; and on the other hand, they will develop a new role of scavenging cell debris, promoting angiogenesis and growth and proliferation of cancer cells [9,44]. One limitation of our study was that the sample size was not big enough.

## Conclusions

This study is the first to analyze the expression of CD163 as a marker of M2 macrophages in a TMA set of LSCCs and correlate that expression with the clinical pathology attributes of the study sample. Using a TMA presents the opportunity to reduce the selection bias associated with the use of full histopathology slides. The above paints a picture of a marker that is involved in multiple points along the carcinogenesis pathway, with evidence of significant expression in LSCC tumor cells, and shows that this expression is well correlated with tumor grade, clinical stage, and TNM staging. Because of the above-mentioned highly versatile role of this marker in oncogenesis, attempting to reverse the effect of TAMs in cancer, in general, would, in theory, yield significant results in cancer management. Thus, further in-depth research is needed to elucidate the exact role of CD163+ TAMs in LSCC and find a therapeutic agent that may reverse the effect of these cells.
